# On a Class of Two-Dimensional Douglas and Projectively Flat Finsler Metrics

**DOI:** 10.1155/2013/291491

**Published:** 2013-10-22

**Authors:** Guojun Yang

**Affiliations:** Department of Mathematics, Sichuan University, Chengdu 610064, China

## Abstract

We study a class of two-dimensional Finsler metrics defined by a Riemannian metric **α** and a 1-form **β**. We characterize those metrics which are Douglasian or locally projectively flat by some equations. In particular, it shows that the known fact that **β** is always closed for those metrics in higher dimensions is no longer true in two-dimensional case. Further, we determine the local structures of two-dimensional (**α**, **β**)-metrics which are Douglasian, and some families of examples are given for projectively flat classes with **β** being not closed.

## 1. Introduction

Projective Finsler geometry studies equivalent Finsler metrics on the same manifold with the same geodesics as points [1]. Douglas curvature (**D**) is an important projective invariants in projective Finsler geometry. A Finsler metric is called * Douglasian* if **D** = 0 and * locally projectively flat* if at every point, there are local coordinate systems in which geodesics are straight. It is known that a locally projectively flat Finsler metric can be characterized by **D** = 0 and vanishing Weyl curvature. As we know, the locally projectively flat class of Riemannian metrics is very limited, nothing but the class of constant sectional curvature (Beltrami theorem). However, the class of locally projectively flat Finsler metrics is very rich. It is known that locally projectively flat Finsler metrics must be Douglasian, but Douglas metrics are not necessarily locally projectively flat. Therefore, it is a natural problem to study and classify Finsler metrics which are Douglasian or locally projectively flat. For this problem, we can only investigate some special classes of Finsler metrics.

In this paper, we shall consider a special class of Finsler metrics defined by a Riemannian metric $\alpha =\sqrt{a_{ij}(x)y^{i}y^{j}}$ and a 1-form *β* = *b*_*i*_(*x*)*y* ^*i*^ on a manifold *M*. Such metrics are called (*α*, *β*)-metrics. An (*α*, *β*)-metric can be expressed in the following form:

(1) $$\begin{array}{c} F=\alpha \phi \left(s\right),\;s=\frac{\beta }{\alpha }, \end{array}$$

where *ϕ*(*s*) > 0 is a *C* ^*∞*^ function on (−*b*_*o*_, *b*_*o*_). It is known that *F* is a regular Finsler metric (defined on the whole *TM* − {0} and positive definite) for any (*α*, *β*) with ||*β*||_*α*_ < *b*_*o*_ if and only if

(2) $$\begin{array}{c} \;\;\;\;\phi \left(s\right)-s\phi^{\prime }\left(s\right)+\left(\rho^{2}-s^{2}\right)\phi^{\prime \prime }\left(s\right)>0,\;\left(\left|s\right|\leq \rho <b_{o}\right), \end{array}$$

where *b*_*o*_ is a constant. If *ϕ* does not satisfy (2), then *F* = *αϕ*(*β*/*α*) is singular.

Randers metrics are a special class of (*α*, *β*)-metrics. It is known that a Randers metric *F* = *α* + *β* is a Douglas metric if and only if *β* is closed [2], and it is locally projectively flat if and only if *α* is locally projectively flat and *β* is closed [2, 3]. Usually we call *F* = *αϕ*(*s*) with $\phi \left(s\right)=\epsilon s+\sqrt{1+ks^{2}}$, where *k*, *ϵ* are constants, a Finsler metric of * Randers type*, which is essentially a Randers metric.

(*α*, *β*)-metrics are computable, and it has been shown that (*α*, *β*)-metrics have a lot of special geometric properties [4–13]. In [5, 10], the authors study and characterize (*α*, *β*)-metrics which are, respectively, Douglasian and locally projectively flat in dimension *n* ≥ 3. However, the two-dimensional case remains open. In this paper, we will solve this problem in two-dimensional case and meanwhile give their local structures in part.

**Theorem 1**

Let *F* = *αϕ*(*s*), *s* = *β*/*α*, be a regular (*α*, *β*)-metric on an open subset *U* ⊂ *R* ^2^, where *ϕ*(0) = 1. Suppose that *β* is not parallel with respect to *α* and *F* is not of Randers type. Let *F* be a Douglas metric. Then one has one of the following two cases.

(i) *ϕ*(*s*) satisfies
  (3) {1+(k1+k3)s2+k2s4}ϕ′′(s)  =(k1+k2s2){ϕ(s)−sϕ′(s)},
  where *k*_1_, *k*_2_, and *k*_3_ are constants satisfying
  (4) $$\begin{array}{c} k_{2}\neq \frac{\left(2k_{1}+3k_{3}\right)\left(3k_{1}+2k_{3}\right)}{25},\;k_{2}\neq k_{1}k_{3}. \end{array}$$
  Further, *β* must be closed.
(ii) *F* can be written as
  (5) $$\begin{array}{c} F=\tilde{\alpha }\pm \frac{{\tilde{\beta }}^{2}}{\tilde{\alpha }},\;\left(\tilde{\alpha }:=\sqrt{\alpha^{2}-k\beta^{2}},\;\;\tilde{\beta }:=c\beta \right), \end{array}$$
  where *k*, *c* are constants with *c* ≠ 0. In this case, *β* is generally not closed.

In dimension *n* > 2 in Theorem 1, it is proved in [5, 10] that the metric in Theorem 1 must be given by (3) with *k*_2_ ≠ *k*_1_ *k*_3_ and *β* must be closed. In Theorems 9 and 11, we give general characterizations for two-dimensional (*α*, *β*)-metrics (might be singular) which are Douglasian and locally projectively flat, respectively.

 Next we consider the local structure of the Douglas metrics in Theorem 1. By using some deformations on *α* and *β*, we can determine the local structure of two-dimensional regular Douglas (*α*, *β*)-metrics, which is shown in the following two theorems. For the local structure of the singular Douglas classes in Theorem 9(iii) and Theorem 9(iv), we will have a discussion in Section 6.

**Theorem 2**

Let *F* = *α* ± *β* ^2^/*α* be a two-dimensional regular Douglas (*α*, *β*)-metric with *β* ≠ 0. Then *α* and *β* can be locally written as

(6) α2=1(1∓B)3×{Bu2+v2[(y1)2+(y2)2]∓9(1±B+B2)β2},

(7) $$\begin{array}{c} \beta =\frac{B}{{\left(1\pm 2B\right)}^{3/2}}\frac{uy^{1}+vy^{2}}{u^{2}+v^{2}}, \end{array}$$

where *u* = *u*(*x*), *v* = *v*(*x*) are scalar functions such that

(8) $$\begin{array}{c} f\left(z\right)=u+iv,\;z=x^{1}+ix^{2}, \end{array}$$

is a complex analytic function and *B* = *B*(*x*) is a scalar function satisfying 0 < *B* < 1 if *F* = *α* + *β* ^2^/*α* and 0 < *B* < 1/2 if *F* = *α* − *β* ^2^/*α*.

We see that in Theorem 2 the metric is determined by the triple parametric scalar functions (*B*, *u*, *v*), where *u* and *v* are a pair of complex conjugate functions. We will prove Theorem 2 by using Corollary 10 and the result in [14] (also see [15]).

**Theorem 3**

Let *F* = *αϕ*(*s*), *s* = *β*/*α*, be a two-dimensional regular Douglas (*α*, *β*)-metric with *β* ≠ 0, where *ϕ*(*s*) satisfies (3) and (4) and *ϕ*(0) = 1. Then *α* and *β* can be locally written as

(9) $$\begin{array}{c} \alpha =\frac{\sqrt{B}}{c}\sqrt{\frac{{\left(y^{1}\right)}^{2}+{\left(y^{2}\right)}^{2}}{u^{2}+v^{2}}},\;\;\beta =\frac{B\left(uy^{1}+vy^{2}\right)}{c\left(u^{2}+v^{2}\right)},\;\;\;\\ \left(c:=e^{\int_{0}^{B}(1/2)(\left(k_{3}+k_{2}t\right)/\left(1+(k_{1}+k_{3})t+k_{2}t^{2}\right))dt}\right), \end{array}$$

where *B* = *B*(*x*) > 0,  *u* = *u*(*x*), *v* = *v*(*x*) are some scalar functions which satisfy the following PDEs:

(10) $$\begin{array}{c} u_{1}=v_{2},\;\;u_{2}=-v_{1},\;\;v_{1}+\sigma_{1}v=u\sigma_{2}, \end{array}$$

where *u*_*i*_ : = *u*_*x*^*i*^_, *v*_*i*_ : = *v*_*x*^*i*^_, and *σ*_*i*_ : = *σ*_*x*^*i*^_, and *σ* is defined by

(11) $$\begin{array}{c} e^{2\sigma }:=\frac{B}{c^{2}\left(u^{2}+v^{2}\right)}. \end{array}$$

We will prove Theorem 3 by (32) and the result in [14] (also see [15]). The metric in Theorem 3 is determined by the triple parametric scalar functions (*B*, *u*, *v*) which satisfy (10). It seems hard to obtain the complete solutions of the PDEs (10). However, we can give some special solutions of (10). For example, the following triple is a solution:

(12) $$\begin{array}{c} \sigma =x^{1},\;\;u=\left(c_{2}\sin x^{2}-c_{1}\cos x^{2}\right)e^{-x^{1}},\;\;\\ v=\left(c_{1}\sin x^{2}+c_{2}\cos x^{2}\right)e^{-x^{1}}, \end{array}$$

where *c*_1_,  *c*_2_ are constants, and then *B* is determined by (11). 

 Now we consider the local structure of the locally projectively flat metrics in Theorem 1. The local structure of *F* determined by (3) with *k*_2_ ≠ *k*_1_ *k*_3_ (for the dimension *n* ≥ 2) has been solved in [16] (also see another way in [13]). However, it seems difficult to determine the local structure of *F* determined by (5). By using (40) and (98) with *τ* = 0, we can construct the following example, which can be directly verified. We omit the details.

**Example 4**

Let *F* = *α* ± *β* ^2^/*α* be a two-dimensional (*α*, *β*)-metric. Suppose *α* and *β* take the form

(13) $$\begin{array}{c} \alpha =e^{\sigma (x)}\sqrt{{\left(y^{1}\right)}^{2}+{\left(y^{2}\right)}^{2}},\;\;\beta =e^{\sigma (x)}\left(\xi \left(x\right)y^{1}+\eta \left(x\right)y^{2}\right), \end{array}$$

where *ξ* = *ξ*(*x*), *η* = *η*(*x*), *σ* = *σ*(*x*) are some scalar functions. Define

(14) $$\begin{array}{c} \xi \left(x\right)=\pm \frac{1}{\sqrt{2}}\frac{x^{2}+c_{2}}{\sqrt{c_{3}\mp {\left(x^{1}+c_{1}\right)}^{2}\mp {\left(x^{2}+c_{2}\right)}^{2}}},\\ \eta \left(x\right)=\mp \frac{1}{\sqrt{2}}\frac{x^{1}+c_{1}}{\sqrt{c_{3}\mp {\left(x^{1}+c_{1}\right)}^{2}\mp {\left(x^{2}+c_{2}\right)}^{2}}},\\ \sigma \left(x\right)=\ln\left[c_{3}\mp {\left(x^{1}+c_{1}\right)}^{2}\mp {\left(x^{2}+c_{2}\right)}^{2}\right]+c_{4}, \end{array}$$

where *c*_1_, *c*_2_, and *c*_3_ are constants with *c*_3_ > 0. Then *F* is projectively flat with *β* being not closed.

For the singular projectively flat classes in Theorem 11(iii) and Theorem 11(iv), we also construct some examples with *β* being not closed (see Examples 15 and 16 below). As we have shown, it seems an obstacle to determine the local structure of the projectively flat classes when *β* is not closed.

**Open Problem**

Determine the local structure of a two-dimensional (*α*, *β*)-metric *F* = *α* ± *β* ^2^/*α* which is locally projectively flat.

## 2. Preliminaries

Let *F* = *F*(*x*, *y*) be a Finsler metric on an *n*-dimensional manifold *M*. The geodesic coefficients are defined by

(15) $$\begin{array}{c} G^{i}:=\frac{1}{4}g^{il}\left\{{\left[F^{2}\right]}_{x^{k}y^{l}}y^{k}-{\left[F^{2}\right]}_{x^{l}}\right\}. \end{array}$$

A Finsler metric *F* = *F*(*x*, *y*) is called a Douglas metric if the spray coefficients *G* ^*i*^ are in the following form:

(16) $$\begin{array}{c} G^{i}=\frac{1}{2}\Gamma_{jk}^{i}\left(x\right)y^{j}y^{k}+P\left(x,y\right)y^{i}, \end{array}$$

where Γ_*jk*_^*i*^(*x*) are local functions on *M* and *P*(*x*, *y*) is a local positively homogeneous function of degree one in *y*. It is easy to see that *F* is a Douglas metric if and only if *G* ^*i*^ *y* ^*j*^ − *G* ^*j*^ *y* ^*i*^ is a homogeneous polynomial in (*y* ^*i*^) of degree three, which by (16) can be written as [2]

(17) $$\begin{array}{c} G^{i}y^{j}-G^{j}y^{i}=\frac{1}{2}\left(\Gamma_{kl}^{i}y^{j}-\Gamma_{kl}^{j}y^{i}\right)y^{k}y^{l}. \end{array}$$

According to G. Hamel's result, a Finsler metric *F* is projectively flat in *U* if and only if

(18) $$\begin{array}{c} F_{x^{m}y^{l}}y^{m}-F_{x^{l}}=0. \end{array}$$

The above formula implies that *G* ^*i*^ = *Py* ^*i*^ with *P* given by

(19) $$\begin{array}{c} P=\frac{F_{x^{m}}y^{m}}{2F}. \end{array}$$

For a Riemannian metric $\alpha =\sqrt{a_{ij}y^{i}y^{j}}$ and a 1-form *β* = *b*_*i*_ *y* ^*i*^ on a manifold *M*, let ∇*β* = *b*_*i*∣*j*_ *y* ^*i*^ *dx* ^*j*^ denote the covariant derivatives of *β* with respect to *α*. Put

(20) $$\begin{array}{c} r_{ij}:=\frac{1}{2}\left(b_{i\;|\;j}+b_{j\;|\;i}\right),\;\;s_{ij}:=\frac{1}{2}\left(b_{i\;|\;j}-b_{j\;|\;i}\right),\;\\ r_{j}:=b^{i}r_{ij},\;\;\;s_{j}:=b^{i}s_{ij},\;\;s^{i}:=a^{ik}s_{k}, \end{array}$$

where *b* ^*i*^ : = *a* ^*ij*^ *b*_*j*_ and (*a* ^*ij*^) is the inverse of (*a*_*ij*_). 

Consider an (*α*, *β*)-metric *F* = *αϕ*(*β*/*α*). By (15), the spray coefficients *G* ^*i*^ of *F* are given by [3, 4, 8, 10, 11]

(21) Gi=Gαi+αQs0i+α−1Θ(−2αQs0+r00)yi +Ψ(−2αQs0+r00)bi,

where *s*_*j*_^*i*^ = *a* ^*ik*^ *s*_*kj*_, *s*_0_^*i*^ = *s*_*k*_^*i*^ *y* ^*k*^, *s*_*i*_ = *b* ^*k*^ *s*_*ki*_, and *s*_0_ = *s*_*i*_ *y* ^*i*^, and

(22) $$\begin{array}{c} Q:=\frac{\phi^{\prime }}{\phi -s\phi^{\prime }},\\ \Theta :=\frac{Q-sQ^{\prime }}{2\Delta },\\ \Psi :=\frac{Q^{\prime }}{2\Delta },\\ \Delta :=1+sQ+\left(b^{2}-s^{2}\right)Q^{\prime }. \end{array}$$

By (21) one can see that *F* = *αϕ*(*β*/*α*) is a Douglas metric if and only if

(23) αQ(s0iyj−s0jyi)+Ψ(−2αQs0+r00)(biyj−bjyi)  =12(Gkliyj−Gkljyi)ykyl,

where *G*_*kl*_^*i*^ : = Γ_*kl*_^*i*^ − *γ*_*kl*_^*i*^, Γ_*kl*_^*i*^ are given in (16) and *γ*_*kl*_^*i*^ : = ∂^2^ *G*_*α*_^*i*^/∂*y* ^*k*^∂*y* ^*l*^.

Further, *F* = *αϕ*(*β*/*α*) is projectively flat on *U* ⊂ *R* ^*n*^ if and only if

(24) (amlα2−ymyl)Gαm+α3Qsl0 +Ψα(−2αQs0+r00)(αbl−syl)=0,

where *y*_*l*_ = *a*_*ml*_ *y* ^*m*^.

## 3. Equations in a Special Coordinate System

 In order to prove Theorems 9 and 11, one has to simplify (23) and (24). The main technique is to fix a point and choose a special coordinate system (*s*, *y* ^*a*^) as in [10, 11].

Fix an arbitrary point *x* ∈ *M*, and take an orthonormal basis {*e*_*i*_} at *x* such that

(25) $$\begin{array}{c} \alpha =\sqrt{\sum_{i=1}^{n}{\left(y^{i}\right)}^{2}},\;\beta =by^{1}. \end{array}$$

Then we change coordinates (*y* ^*i*^) to (*s*, *y* ^*a*^) such that

(26) $$\begin{array}{c} \alpha =\frac{b}{\sqrt{b^{2}-s^{2}}}\overset{-}{\alpha },\;\;\beta =\frac{bs}{\sqrt{b^{2}-s^{2}}}\overset{-}{\alpha }, \end{array}$$

where $\overset{-}{\alpha }=\sqrt{\sum_{a=2}^{n}{\left(y^{a}\right)}^{2}}$. Let

(27) $$\begin{array}{c} {\overset{-}{r}}_{10}:=r_{1a}y^{a},\;\;{\overset{-}{r}}_{00}:=r_{ab}y^{a}y^{b},\;\;{\overset{-}{s}}_{0}:=s_{a}y^{a}. \end{array}$$

We have ${\overset{-}{s}}_{0}=b{\overset{-}{s}}_{10},s_{1}=bs_{11}=0$. The following lemmas are trivial.

**Lemma 5**

In the special local coordinate system at *x* as mentioned previously, if *b* = *constant*, then *r*_11_ = 0, *r*_1*a*_ + *s*_1*a*_ = 0 at *x*.

**Lemma 6 (see [11])**

For *n* ≥ 2, suppose $p+q\overset{-}{\alpha }=0$, where $p=p(\overset{-}{y})$ and $q=q(\overset{-}{y})$ are homogeneous polynomials in $\overset{-}{y}=(y^{a})$; then *p* = 0, *q* = 0.

The following two propositions are simple corollaries from [5, 10] (also see (23) and (24)).

**Proposition 7 (*n* = 2)**

An (*α*, *β*)-metric *F* = *αϕ*(*β*/*α*) is a Douglas metric if and only if, at each point *x*, there is a suitable coordinate system such that at *x* there exist numbers *G*_*jk*_^*i*^  (*i*, *j*, *k* = 1,2) which are independent of *s* such that

(28) s22(b2−s2)(G111−G122−G212)+12G221  =bΨ(s2b2−s2r11+r22),

(29) 1b2−s2[2Ψ(b2−s2)−1]b3Qs12−2bΨr12s =G1122(b2−s2)s3+12(G222−G121−G211)s.

**Proposition 8 (*n* = 2)**

An (*α*, *β*)-metric *F* = *αϕ*(*β*/*α*) is projectively flat if and only if

(30) $$\begin{array}{c} \frac{s^{2}}{2\left(b^{2}-s^{2}\right)}\left(-{\tilde{G}}_{11}^{1}+2{\tilde{G}}_{12}^{2}\right)-\frac{1}{2}{\tilde{G}}_{22}^{1}=b\Psi \left(\frac{s^{2}}{b^{2}-s^{2}}r_{11}+r_{22}\right), \end{array}$$

(31) 1b2−s2[2Ψ(b2−s2)−1]b3Qs12−2bΨr12s =−G~1122(b2−s2)s3+12(−G~222+2G~121)s,

where ${\tilde{G}}_{jk}^{i}:=\partial^{2}G_{\alpha }^{i}/\partial y^{j}\partial y^{k}$ are the connection coefficients of *α*.

Comparing (28) and (30), (29) and (31), it is easy to see that if *G*_*jk*_^*i*^ = *G*_*kj*_^*i*^, then ${\tilde{G}}_{jk}^{i}=-G_{jk}^{i}$. So if we can solve *G*_*jk*_^*i*^ from (28) and (29), then we can solve ${\tilde{G}}_{jk}^{i}$ from (30) and (31). In the following we only consider (28) and (29), from which we will solve *G*_*jk*_^*i*^.

## 4. Douglas (*α*, *β*)-Metrics

In this section, we characterize two-dimensional (*α*, *β*)-metrics (might be singular) which are Douglas metrics. We have the following theorem.

**Theorem 9**

Let *F* = *αϕ*(*s*), *s* = *β*/*α*, be an (*α*, *β*)-metric on an open subset *U* ⊂ *R* ^2^, where *ϕ*(0) = 1. Suppose that *β* is not parallel with respect to *α* and *F* is not of Randers type. Then *F* is a Douglas metric on *U* if and only if *F* lies in one of the following four classes:

(i) *ϕ*(*s*) satisfy (3) with *k*_2_ ≠ *k*_1_ *k*_3_ and *β* satisfies
  (32) $$\begin{array}{c} b_{i\;|\;j}=2\tau \left\{\left(1+k_{1}b^{2}\right)a_{ij}+\left(k_{2}b^{2}+k_{3}\right)b_{i}b_{j}\right\}, \end{array}$$
  (ii) *ϕ*(*s*) and *β* satisfy
  (33) $$\begin{array}{c} \phi \left(s\right)=\sqrt{1-ks^{2}}+\frac{cs^{2}}{\sqrt{1-ks^{2}}}, \end{array}$$
  (34) rij=2τ{[1+(2c−k)b2]aij−[k+3c−(k+c)kb2]bibj} +d(bisj+bjsi),
  where *τ* = *τ*(*x*) is a scalar function, *k*, *c* are constants with *c* ≠ 0 and 1 − *kb* ^2^ ≥ 0, and *d* = *d*(*x*) is given by
  (35) $$\begin{array}{c} d=\frac{3c-k-\left(2c-k\right)kb^{2}}{1-\left(k+c\right)b^{2}}. \end{array}$$
(iii) (*b* = constant) *ϕ*(*s*) and *β* satisfy
  (36) $$\begin{array}{c} \phi \left(s\right)=\frac{\sqrt{b^{2}-s^{2}}}{b}+\sqrt{b^{2}-s^{2}}\int_{0}^{s}\frac{c}{{\left(b^{2}-t^{2}\right)}^{3/2}}{\left(\frac{t^{2}}{1-kt^{2}}\right)}^{m}dt, \end{array}$$
  (37) $$\begin{array}{c} r_{ij}=-\frac{1}{b^{2}}\left(b_{i}s_{j}+b_{j}s_{i}\right), \end{array}$$
  where *c*, *k* are constants and *m* ≥ 1 is an integer.
(iv) (*b* = constant) *ϕ*(*s*) and *β* satisfy
  (38) ϕ(s)=b2−s2b+b2−s2∫0sc(b2−t2)3/2(t21−kt2)m−1/2dt,
  (39) $$\begin{array}{c} r_{ij}=-\frac{1}{b^{2}}\left(b_{i}s_{j}+b_{j}s_{i}\right), \end{array}$$
  where *c*, *k* are constants and *m* ≥ 1 is an integer.

By Theorem 9(ii), we can easily get the following corollary.

**Corollary 10**

Let *F* = *α* ± *β* ^2^/*α* be a two-dimensional (*α*, *β*)-metric. Then *F* is a Douglas metric if and only if *β* satisfies

(40) $$\begin{array}{c} r_{ij}=2\tau \left\{\left(1\pm 2b^{2}\right)a_{ij}\mp 3b_{i}b_{j}\right\}+\frac{3}{\pm 1-b^{2}}\left(b_{i}s_{j}+b_{j}s_{i}\right), \end{array}$$

where *τ* = *τ*(*x*) is a scalar function. Note that *F* = *α* + *β* ^2^/*α* is regular if and only if *b* < 1; *F* = *α* − *β* ^2^/*α* is regular if and only if *b* < 1/2.

We prove Theorem 9 using Proposition 7. The proof can be divided into two cases (*r*_11_, *r*_22_)≠(0,0) and (*r*_11_, *r*_22_) = (0,0).

### 4.1. (*r*_11_, *r*_22_)≠(0,0)

In this case, we will obtain two classes: Theorem 9(i) and Theorem 9(ii).

First, (28) can be written in the following form:

(41) $$\begin{array}{c} 2\Psi =\frac{\lambda s^{2}+\mu \left(b^{2}-s^{2}\right)}{\delta s^{2}+\eta \left(b^{2}-s^{2}\right)}, \end{array}$$

where *λ*, *μ*, *δ*, and *η* are numbers independent of *s*. By (28) and (41), it is easy to prove that if *λη* − *μδ* ≠ 0, then for some scalar *τ* = *τ*(*x*), we have (see also [5])

(42) $$\begin{array}{c} r_{11}=2b^{2}\delta \tau ,\;\;r_{22}=2b^{2}\eta \tau . \end{array}$$

One can see that if an (*α*, *β*)-metric *F* = *αϕ*(*β*/*α*) is not of Randers type, then *λη* − *μδ* ≠ 0.

Now we put

(43) $$\begin{array}{c} a_{0}:=1,\;\;a_{i}:=\frac{\phi^{\left(i\right)}\left(0\right)}{i!},\;i=1,2,\ldots . \end{array}$$

By (41), it has been proved in [10] that if 2*a*_4_ + *a*_2_^2^ = 0, then *F* is of Randers type. Thus we may assume that 2*a*_4_ + *a*_2_^2^ ≠ 0. Then there is a scalar *ϵ* = *ϵ*(*x*) ≠ 0 such that

(44) μ=k1ϵ,  η=(1+k1b2)ϵ,  λ=(k1+k2b2)ϵ,  δ=(1+(k1+k3)b2+k2b4)ϵ,

(45) 2Ψ=k1+k2s21+k1b2+(k3+k2b2)s2,

where *k*_1_, *k*_2_, and *k*_3_ are some constants determined by

(46) $$\begin{array}{c} k_{1}=2a_{2},\;\;k_{2}=\frac{2\left(a_{4}a_{2}^{2}-5a_{2}a_{6}+12a_{4}^{2}\right)}{2a_{4}+a_{2}^{2}},\\ k_{3}=-\frac{11a_{2}a_{4}+5a_{6}+3a_{2}^{3}}{2a_{4}+a_{2}^{2}}. \end{array}$$

Note that *k*_2_ − *k*_1_ *k*_3_ ≠ 0 is equivalent to 2*a*_4_ + *a*_2_^2^ ≠ 0. Since *F* is not of Randers type, we get *k*_2_ − *k*_1_ *k*_3_ ≠ 0.

Plugging (45) into (29), we get

(47) −2bs(b2−s2)(k1+k2s2)r12−2b3(1+k1s2+k3s2+k2s4)Qs12+s(1+k1b2+k3s2+k2b2s2)×{(b2−s2)ξ−G112s2}=0,

where *ξ* : = *G*_12_^1^ + *G*_21_^1^ − *G*_22_^2^. Now plug the Taylor expansion of *ϕ*(*s*) into (47), and let *p*_*i*_ be the coefficients of *s* ^*i*^ in (47). By *p*_1_ = 0, *p*_3_ = 0, and *p*_5_ = 0, we have the following cases.

(i) If

(48) $$\begin{array}{c} 1+\left(k_{1}+k_{3}\right)b^{2}+k_{2}b^{4}\neq 0, \end{array}$$

then

(49) r12=b215(k1(3k12+2k1k3+10k2)b4 +(21k12+14k1k3−5k2)b2 +18k1−3k3)×(1+(k1+k3)b2+k2b4)−1s12,

(50) G112=2b15((3k12k3+2k1k32−2k2k3−18k1k2)b4 −5(3k12+k2+2k1k3)b2−15k1)×(1+(k1+k3)b2+k2b4)−1s12,

(51) ξ=2bk115((3k12+2k1k3+10k2)b4  +6(3k1+2k3)b2+15)×(1+(k1+k3)b2+k2b4)−1s12.

(ii) If
  (52) $$\begin{array}{c} 1+\left(k_{1}+k_{3}\right)b^{2}+k_{2}b^{4}=0, \end{array}$$

then *b* = constant. By Lemma 5 we get *r*_12_ + *s*_12_ = 0. If *s*_12_ ≠ 0, then we get

(53) $$\begin{array}{c} k_{2}=\frac{3+k_{1}b^{2}}{2b^{4}},\;\;k_{3}=-\frac{5+3k_{1}b^{2}}{2b^{2}}. \end{array}$$

**Case 1**

*s*_12_ = 0. This implies Theorem 9(i).

If (48) holds, then *r*_12_ = 0 by (49). If (52) holds, we also get *r*_12_ = 0. In both cases, we have *G*_11_^2^ = *ξ* = 0 by plugging *r*_12_ = *s*_12_ = 0 into (29). Thus (29) becomes trivial. By *r*_12_ = 0, (42) and (44), we get the expression of *b*_*i*∣*j*_ in (32). Further, (45) can be written in the form (3) with *k*_2_ ≠ *k*_1_ *k*_3_. This class belongs to Theorem 9(i).

**Case**

*s*_12_ ≠ 0. This implies Theorem 9(ii).

*Case 2A*. Assume that (48) holds. We plug (49), (50), and (51) into (47), and then we obtain

(54) Q=−115{(3k12k3+2k1k32−2k2k3−18k1k2)s4  −5(3k12+k2+2k1k3)s2−15k1}s ×(1+(k1+k3)s2+k2s4)−1.

By (54) and (45), we have

(55) $$\begin{array}{c} k_{2}=\frac{\left(2k_{1}+3k_{3}\right)\left(3k_{1}+2k_{3}\right)}{25}. \end{array}$$

Plug (55) into (54) and we get

(56) $$\begin{array}{c} \phi \left(s\right)=\frac{1}{\sqrt{5}}\frac{5+\left(4k_{1}+k_{3}\right)s^{2}}{\sqrt{5+\left(3k_{1}+2k_{3}\right)s^{2}}}, \end{array}$$

where *k*_1_ ≠ *k*_3_ since (55) and *k*_2_ ≠ *k*_1_ *k*_3_. Letting *k*_1_ = 2*c* − *k* and *k*_3_ = −3*c* − *k* in (56), we get (33). Substituting (55) into (49) gives

(57) $$\begin{array}{c} r_{12}=\frac{b^{2}\left[k_{1}\left(3k_{1}+2k_{3}\right)b^{2}+6k_{1}-k_{3}\right]}{5+\left(2k_{1}+3k_{3}\right)b^{2}}s_{12}. \end{array}$$

Letting *k*_1_ = 2*c* − *k* and *k*_3_ = −3*c* − *k* and using (42), we obtain (34).

*Case 2B*. Assume that (52) holds. Then *r*_12_ = −*s*_12_ and (53) holds. It is easy for us to get

(58) $$\begin{array}{c} \phi \left(s\right)=\frac{b+\tilde{c}s^{2}}{\sqrt{b^{2}-s^{2}}}, \end{array}$$

(59) $$\begin{array}{c} r_{ij}=2\tilde{\tau }\left(b^{2}a_{ij}-b_{i}b_{j}\right)-\frac{1}{b^{2}}\left(b_{i}s_{j}+b_{j}s_{i}\right). \end{array}$$

This class is a special case of Theorem 1(ii).

### 4.2. (*r*_11_, *r*_22_)  =  (0,0)

Since *β* is not parallel and (*r*_11_, *r*_22_) = (0,0), we will see that *s*_12_ ≠ 0 from the following proof to different cases. It follows from (28) that

(60) $$\begin{array}{c} G_{22}^{1}=0,\;\;G_{11}^{1}=G_{12}^{2}+G_{21}^{2}. \end{array}$$

Plugging the expressions of *Q* and Ψ into (29) yields

(61) s(b2−s2)[2br12+G112s2−(b2−s2)ξ]ϕ′′ +s[G112s2−(b2−s2)ξ](ϕ−sϕ′) +2b3s12ϕ′=0,

where *ξ* : = *G*_12_^1^ + *G*_21_^1^ − *G*_22_^2^.

Let

(62) $$\begin{array}{c} \phi =a_{0}+\sum_{i=1}^{h}a_{i}s^{i}+o\left(s^{h}\right),\;a_{0}=1, \end{array}$$

where *h* is a sufficiently large integer. Plugging the above Taylor series into (61), we obtain a power series ∑_*k*_ *p*_*k*_ *s* ^*k*^ = 0. It is easily seen that the coefficient *p*_*k*_ of *s* ^*k*^ is given by

(63) $$\begin{array}{c} p_{k}=A_{1}r_{12}+A_{2}G_{11}^{2}+A_{3}\xi +A_{4}s_{12}, \end{array}$$

where

(64) A1:=b[(k−1)(k−2)ak−1−k(k+1)ak+1b2],A2:=12(k−2)[(k−4)ak−3−(k−1)ak−1b2],A3:=12[k(k+1)ak+1b4 −(2k−1)(k−2)ak−1b2 +(k−2)(k−4)ak−3],A4:=−(k+1)ak+1b3,

and *a*_*i*_ = 0 if *i* < 0. In particular, we have

(65) $$\begin{array}{c} p_{0}=-a_{1}b^{3}s_{12}. \end{array}$$

So if *s*_12_ ≠ 0, then by *p*_0_ = 0, we have *a*_1_ = 0.

*Case I*. Suppose *db* ≠ 0. We will prove that one case belongs to Theorem 9(ii) with the scalar *τ* = *τ*(*x*) = 0, and other cases are excluded.

Solving the system *p*_1_ = 0, *p*_3_ = 0, *p*_5_ = 0 yields the following three cases.

(i) If 2*a*_4_ ≠ −*a*_2_^2^, then we get (49), (50), and (51) by using (46).
(ii) If 2*a*_4_ = −*a*_2_^2^ and 2*a*_6_ ≠ *a*_2_^3^, then
  (66) $$\begin{array}{c} r_{12}=\frac{1}{5}\left(8a_{2}b^{2}-1\right)s_{12},\;\;G_{11}^{2}=-4a_{2}bs_{12},\\ \xi =\frac{16}{5}a_{2}bs_{12}. \end{array}$$
  (iii) If 2*a*_4_ = −*a*_2_^2^ and 2*a*_6_ = *a*_2_^3^, then
  (67) $$\begin{array}{c} G_{11}^{2}=-4a_{2}bs_{12},\;\;\xi =\frac{4a_{2}b}{1+2a_{2}b^{2}}\left(r_{12}+s_{12}\right). \end{array}$$

It follows from (49) or (66) that *r*_12_ = 0 if *s*_12_ = 0. If (67) holds and *s*_12_ = 0, then we have *G*_11_^2^ = 0, and thus we have *r*_12_ = 0 by (29) since *F* is not of Randers type (also see the proof in [5]). Therefore in this case we have *s*_12_ ≠ 0.

*Case IA*. Suppose 2*a*_4_ = −*a*_2_^2^ and 2*a*_6_ ≠ *a*_2_^3^. Then plug (66) into (61), and by using *db* ≠ 0 we get $\phi (s)=\sqrt{1+2a_{2}s^{2}}$. This case is excluded.

*Case IB*. Suppose *a*_4_ ≠ −(1/2)*a*_2_^2^. Plugging (49), (50), and (51) into (61) and by using *db* ≠ 0 and *s*_12_ ≠ 0, we obtain three ODEs on *ϕ*(*s*) whose discussion of solutions can be divided into the following cases.

If *k*_3_ ≠ *k*_1_, then in a similar way as in Section 4.1, we can easily show that this class belongs to Theorem 1(ii) with *τ* = *τ*(*x*) = 0.

If *k*_3_ = *k*_1_ ≠ 0, then we obtain *k*_2_ = *k*_1_^2^, which is impossible since *k*_2_ ≠ *k*_1_ *k*_3_.

If *k*_1_ = *k*_3_ = 0, then it is easy to see that *F* is of Randers type, which is excluded.

*Case IC*. Suppose *a*_4_ = −(1/2)*a*_2_^2^ and *a*_6_ = (1/2)*a*_2_^3^. Then we have (67). Note that we have *a*_1_ = 0 since *s*_12_ ≠ 0. We will show that this case is excluded.

For the function $f(s)=\sqrt{1+2a_{2}s^{2}}$, its Taylor coefficients *c*_*i*_ of *s* ^*i*^ (*i* ≥ 0) are given by

(68) $$\begin{array}{c} c_{2i+1}=0,\;\;c_{2i}=C_{1/2}^{i}{\left(2a_{2}\right)}^{i}, \end{array}$$

where *C*_*μ*_^*i*^ are the generalized combination coefficients. So in all *a*_2*i*+1_'s or *a*_2*i*_'s there exists some minimal *m* such that

(69) $$\begin{array}{c} a_{2m+1}\neq 0,\;\left(m\geq 1\right),\text{or}\;\;\\ a_{2m}\neq C_{1/2}^{m}{\left(2a_{2}\right)}^{m},\;\left(m\geq 4\right). \end{array}$$

*Case *IC*(1)*. Assume *a*_2*m*+1_ ≠ 0 in (69). Then plugging (67), *a*_2*m*−3_ = 0 and *a*_2*m*−1_ = 0 into *p*_2*m*_ = 0 (see (63)) yields

(70) $$\begin{array}{c} a_{1+2m}\left[-2mr_{12}+\left(4ma_{2}b^{2}-2a_{2}b^{2}-1\right)s_{12}\right]=0. \end{array}$$

Therefore, it follows from (70) that we have

(71) $$\begin{array}{c} r_{12}=\frac{4ma_{2}b^{2}-2a_{2}b^{2}-1}{2m}s_{12}. \end{array}$$

*Case *IC*(2)*. Assume *a*_2*m*_ ≠ *C*_1/2_^*m*^(2*a*_2_)^*m*^ in (69). Plugging (67) and

(72) $$\begin{array}{c} a_{2m-4}=C_{1/2}^{m-2}{\left(2a_{2}\right)}^{m-2},\;\;a_{2m-2}=C_{1/2}^{m-1}{\left(2a_{2}\right)}^{m-1} \end{array}$$

into *p*_2*m*−1_ = 0 (see (63)) yields

(73) [a2m−C1/2m(2a2)m] ×[(1−2m)r12+(4ma2b2−4a2b2−1)s12]=0.

Therefore it follows from (73) that we have

(74) $$\begin{array}{c} r_{12}=\frac{4ma_{2}b^{2}-4a_{2}b^{2}-1}{2m-1}s_{12}. \end{array}$$

Finally, plugging (67) and (71) or (74) into (61) and using *db* ≠ 0 we get $\phi (s)=\sqrt{1+2a_{2}s^{2}}$. Thus both cases are excluded.

*Case II*. Suppose *db* = 0. We will obtain Theorem 9(iii) and Theorem 1(iv).

By Lemma 5 we have

(75) $$\begin{array}{c} r_{12}=-s_{12}. \end{array}$$

For the function $f(s)=\left(1/b\right)\sqrt{b^{2}-s^{2}}$, its Taylor coefficients *c*_*i*_ of *s* ^*i*^ (*i* ≥ 0) are given by

(76) $$\begin{array}{c} c_{2i+1}=0,\;\;c_{2i}=C_{1/2}^{i}{\left(-\frac{1}{b^{2}}\right)}^{i}. \end{array}$$

Since *a*_1_ = 0 and *F* is not of Randers type, in all *a*_2*i*+1_'s or *a*_2*i*_'s, there exists some minimal *m* such that

(77) $$\begin{array}{c} a_{2m+1}\neq 0,\;\left(m\geq 1\right),\text{or}\;\;\\ a_{2m}\neq C_{1/2}^{m}{\left(-\frac{1}{b^{2}}\right)}^{m},\;\left(m\geq 1\right). \end{array}$$

Case *II(1)*. Assume *a*_2*m*+1_ ≠ 0 in (77). Plugging (75), *a*_2*m*−3_ = 0 and *a*_2*m*−1_ = 0 into *p*_2*m*_ = 0 (see (63)) yields

(78) $$\begin{array}{c} \xi =-\frac{2m-1}{mb}s_{12}. \end{array}$$

Plugging (78) and *a*_2*m*−1_ = 0 into *p*_2*m*+2_ = 0 yields

(79) $$\begin{array}{c} G_{11}^{2}=\frac{1}{m\left(2m+1\right)}\left[\frac{\left(2m+3\right)ba_{2m+3}}{ma_{2m+1}}+\frac{4m^{2}-3}{b}\right]s_{12}. \end{array}$$

Now plug (75), (78), and (79) into (61), and then we obtain

(80) $$\begin{array}{c} \frac{\phi -s\phi^{\prime }+\left(b^{2}-s^{2}\right)\phi^{\prime \prime }}{s\phi +\left(b^{2}-s^{2}\right)\phi^{\prime }}=\frac{2m}{s\left(1-ks^{2}\right)}, \end{array}$$

where *k* is a constant determined by *a*_2*m*+1_ and *a*_2*m*+3_. Let

(81) $$\begin{array}{c} \Phi :=s\phi \left(s\right)+\left(b^{2}-s^{2}\right)\phi^{\prime }\left(s\right). \end{array}$$

Then (80) becomes

(82) $$\begin{array}{c} \frac{\Phi^{\prime }}{\Phi }=\frac{2m}{s\left(1-ks^{2}\right)}. \end{array}$$

We get

(83) $$\begin{array}{c} \Phi =c{\left(\frac{s^{2}}{1-ks^{2}}\right)}^{m}, \end{array}$$

where *c* is a constant. Then we can easily get

(84) $$\begin{array}{c} \phi =\sqrt{b^{2}-s^{2}}\int_{}^{}\frac{c}{{\left(b^{2}-s^{2}\right)}^{3/2}}{\left(\frac{s^{2}}{1-ks^{2}}\right)}^{m}ds. \end{array}$$

By assumption, *ϕ*(0) = 1, we get (36). Further, since *r*_11_ = 0, *r*_22_ = 0, and *r*_12_ = −*s*_12_, we get (37). This class belongs to Theorem 9(iii).

*Case II(2)*. Assume *a*_2*m*_ ≠ *C*_1/2_^*m*^(−1/*b* ^2^)^*m*^ in (77). Plugging (75) and the expressions of *a*_2*m*−4_ and *a*_2*m*−2_ into *p*_2*m*−1_ = 0 yields

(85) $$\begin{array}{c} \xi =-\frac{4\left(m-1\right)}{\left(2m-1\right)b}s_{12}. \end{array}$$

Plugging (85) and the expressions of *a*_2*m*−2_ into *p*_2*m*+1_ = 0 (see (63)) yields

(86) $$\begin{array}{c} G_{11}^{2}=\frac{T_{1}}{T_{2}}s_{12}, \end{array}$$

where *T*_1_ and *T*_2_ are defined by

(87) T1:=4m(2m−1)(m−1)C1/2m(−1b2)m−1+2(2m−1)(2m2−2m−1)b2a2m+4(m+1)b4a2m+2,T2:=m(2m−1)2b3[a2m−C1/2m(−1b2)m].

Now plug (75), (85), and (86) into (61), and then we obtain

(88) $$\begin{array}{c} \frac{\phi -s\phi^{\prime }+\left(b^{2}-s^{2}\right)\phi^{\prime \prime }}{s\phi +\left(b^{2}-s^{2}\right)\phi^{\prime }}=\frac{2m-1}{s\left(1-ks^{2}\right)}, \end{array}$$

where *k* is a constant. By the same argument, we obtain (38). This gives Theorem 9(iv).

## 5. Projectively Flat (*α*, *β*)-Metrics

 In this section, we characterize two-dimensional (*α*, *β*)-metrics (might be singular) which are projectively flat. We have the following theorem.

**Theorem 11**

Let *F* = *αϕ*(*s*), *s* = *β*/*α*, be an (*α*, *β*)-metric on an open subset *U* ⊂ *R* ^2^ with *ϕ*(0) = 1. Suppose that *β* is not parallel with respect to *α* and *F* is not of Randers type. Then *F* is projectively flat in *U* with *G* ^*i*^ = *P*(*x*, *y*)*y* ^*i*^ if and only if *F* lies in one of the following four classes.

(i) *ϕ*(*s*) and *β* satisfy (3) and (32), and the spray coefficients *G*_*α*_^*i*^ of *α* satisfy
  (89) $$\begin{array}{c} G_{\alpha }^{i}=\rho y^{i}-\tau \left(k_{1}\alpha^{2}+k_{2}\beta^{2}\right)b^{i}. \end{array}$$
  In this case, the projective factor *P* is given by
  (90) P=ρ+τα{[1+(k1+k3)s2+k2s4]ϕ′ϕ      −(k1+k2s2)s}.
(ii) *ϕ*(*s*) and *β* satisfy (33) and (34), and
  (91) Gαi=ρyi−τ{(2c−k)α2+(c+k)kβ2}bi +(2c−k)(1−kb2)α2+ckβ21−(c+k)b2si.
  In this case, the projective factor *P* is given by
  (92) $$\begin{array}{c} P=\rho -\frac{4c^{2}s^{3}}{1+\left(c-k\right)s^{2}}\tau \alpha +\frac{\sigma_{1}}{\sigma_{2}}s_{0}, \end{array}$$
  where
  (93) σ1:=[(k−2c)(c−k)kb2+4c2−3kc+k2]s2+(2c−k)(1−kb2),σ2:=[1−(c+k)b2][1+(c−k)s2].
(iii) *ϕ*(*s*) and *β* satisfy (36) and (37), and
  (94) $$\begin{array}{c} G_{\alpha }^{i}=\rho y^{i}-\frac{\left(2m-1\right)\alpha^{2}+k\beta^{2}}{2mb^{2}}s^{i}. \end{array}$$
  In this case, the projective factor *P* is given by
  (95) $$\begin{array}{c} P=\rho -\frac{s\left(1-ks^{2}\right)\phi^{\prime }+\left[\left(2m-1\right)+ks^{2}\right]\phi }{2mb^{2}\phi }s_{0}. \end{array}$$
(iv) *ϕ*(*s*) and *β* satisfy (38) and (39), and
  (96) $$\begin{array}{c} G_{\alpha }^{i}=\rho y^{i}-\frac{2\left(m-1\right)\alpha^{2}+k\beta^{2}}{\left(2m-1\right)b^{2}}s^{i}. \end{array}$$
  In this case, the projective factor *P* is given by
  (97) $$\begin{array}{c} P=\rho -\frac{s\left(1-kb^{2}\right)\phi^{\prime }+\left[2\left(m-1\right)+ks^{2}\right]\phi }{\left(2m-1\right)b^{2}\phi }s_{0}. \end{array}$$

In the above, *ρ* = *c*_1_(*x*)*y* ^1^ + *c*_2_(*x*)*y* ^2^ is a 1-form.

By Theorem 11(ii), we can easily get the following corollary.

**Corollary 12**

Let *F* = *α* ± *β* ^2^/*α* be two-dimensional (*α*, *β*)-metric. Then *F* is locally projectively flat if and only if *β* satisfies (40) and *G*_*α*_^*i*^ satisfy

(98) $$\begin{array}{c} G_{\alpha }^{i}=\rho y^{i}\mp 2\tau \alpha^{2}b^{i}-\frac{2\alpha^{2}}{b^{2}\mp 1}s^{i}. \end{array}$$

In this case, the projective factor *P* is given by

(99) $$\begin{array}{c} P=\rho -\frac{4s^{3}}{1\pm s^{2}}\tau \alpha -\frac{2\left(2s^{2}\pm 1\right)}{\left(b^{2}\mp 1\right)\left(s^{2}\pm 1\right)}s_{0}. \end{array}$$

To prove Theorem 11, it follows from comparing Propositions 7 and 8 that we only need to give the expressions (89)–(97) for each class in Theorem 11.

### 5.1. The Spray Coefficients of *α*

 In this subsection we will show the expressions of the spray coefficients *G*_*α*_^*i*^ for each class in Theorem 11. Note that by ${\tilde{G}}_{jk}^{i}=\partial^{2}G_{\alpha }^{i}/\partial y^{j}\partial y^{k}$, the spray *G*_*α*_^*i*^ of *α* can be expressed as

(100) $$\begin{array}{c} G_{\alpha }^{i}=\frac{1}{2}{\tilde{G}}_{jk}^{i}y^{j}y^{k}. \end{array}$$

*Case I*. Suppose that (*r*_11_, *r*_22_)≠(0,0). It has been proved in [10] that

(101) $$\begin{array}{c} {\tilde{G}}_{11}^{1}=2t_{1}-2\lambda b^{3}\tau ,\;\;{\tilde{G}}_{22}^{1}=-2\mu b^{3}\tau ,\\ {\tilde{G}}_{12}^{1}=t_{2},\;\;{\tilde{G}}_{12}^{2}=t_{1}, \end{array}$$

where *t*_1_, *t*_2_ are numbers independent of *s* and *τ* is given by (32) or (34). By (50) and (51), we can get ${\tilde{G}}_{11}^{2}$ and ${\tilde{G}}_{22}^{2}$.

If *β* is closed (*s*_12_ = 0), then it follows from (44) and (101) and the expressions of ${\tilde{G}}_{11}^{2}$ and ${\tilde{G}}_{22}^{2}$ that (89) holds, where we put *ρ* as *ρ* = *t*_*i*_ *y* ^*i*^. This case has been given by [10] in case of *n* ≥ 3.

If *β* is not closed, then by putting *k*_1_ = 2*c* − *k* and *k*_3_ = −3*c* − *k* we get (91) from (55) and (101) and the expressions of ${\tilde{G}}_{11}^{2}$ and ${\tilde{G}}_{22}^{2}$.

*Case II*. Suppose that (*r*_11_, *r*_22_) = (0,0). Then by (30) we get

(102) $$\begin{array}{c} {\tilde{G}}_{22}^{1}=0,\;\;{\tilde{G}}_{11}^{1}=2{\tilde{G}}_{12}^{2}=2t_{1},\\ {\tilde{G}}_{12}^{2}=t_{1},\;\;{\tilde{G}}_{12}^{1}=t_{2}. \end{array}$$

If *db* ≠ 0, then we have shown in Section 4.2 that *a*_4_ ≠ −(1/2)*a*_2_^2^. In this case, we obtain (91) with *τ* = 0.

If *db* = 0, then we get (77). If *a*_2*m*+1_ ≠ 0 in (77), then we get from (78), (79) that

(103) $$\begin{array}{c} {\tilde{G}}_{11}^{2}=-\frac{2m-1+kb^{2}}{mb}s_{12},\;\;{\tilde{G}}_{22}^{2}=2{\tilde{G}}_{12}^{1}-\frac{2m-1}{mb}s_{12}. \end{array}$$

Then it follows from (102) and (103) that (94) holds. If *a*_2*m*_ ≠ *C*_1/2_^*m*^(−1/*b* ^2^)^*m*^ in (77), then we get from (85), (86) that

(104) $$\begin{array}{c} {\tilde{G}}_{11}^{2}=-\frac{2\left(2m^{2}-2m-k\right)}{m\left(2m-1\right)b}s_{12},\\ {\tilde{G}}_{22}^{2}=2{\tilde{G}}_{12}^{1}-\frac{4\left(m-1\right)}{\left(2m-1\right)b}s_{12}. \end{array}$$

Then it follows from (102) and (104) that (96) holds.

### 5.2. The Projective Factors

 In this subsection, we are going to find the expression for the projective factor for each class in Theorem 11.

Actually, (90) has been proved in [10], since *β* is closed in Theorem 11(i). So we only show the expressions of *P* in (92), (95), and (97). In the left three classes, since *β* may not be closed, it is not easy to show the projective factors *P* in the initial local projective coordinate system (in such a coordinate system, geodesics are straight lines). However, it is easy to be solved by choosing another local projective coordinate system and then returning to the initial local projective coordinate system, just as that in [10].

Fix an arbitrary point *x*_*o*_ ∈ *U* ⊂ *R* ^2^. By the above idea and a suitable affine transformation, we may assume (*U*, *x* ^*i*^) is a local projective coordinate system satisfying that $\alpha_{x_{o}}=\sqrt{{\left(y^{1}\right)}^{2}+{\left(y^{2}\right)}^{2}}$ and *β*_*x*_*o*__ = *by* ^1^. Then at *x*_*o*_ we have

(105) $$\begin{array}{c} s^{1}=s_{1}=0,\;\;s^{2}=s_{2}=bs_{12},\;\;\;\\ s_{0}=bs_{12}y^{2},\;\;\;b^{1}=b_{1}=b,\;\;b^{2}=b_{2}=0. \end{array}$$

Suppose (33), (34), and (91) hold in *U*. Then it is easy to get *r*_00_, *s*_0_^*i*^, *Q*, Θ, and Ψ. Plug them into (21), and then at *x*_*o*_ we see that *G* ^*i*^ = *Py* ^*i*^, where *P* is given by

(106) $$\begin{array}{c} P=\rho +A_{1}\tau +A_{2}bs_{12}y^{2}, \end{array}$$

where

(107) A1:=−4c2(by1)3[1+(c−k)b2](y1)2+(y2)2,A2:=([1+(2c−k)b2][(2c−k)−k(c−k)b2]×(y1)2+(2c−k)(1−kb2)(y2)2)×([1−(c+k)b2]    ×{[1+(c−k)b2](y1)2+(y2)2})−1.

By using

(108) $$\begin{array}{c} bs_{12}y^{2}=s_{0},\;\;{\left(y^{1}\right)}^{2}+{\left(y^{2}\right)}^{2}=\alpha^{2},\\ by^{1}=\beta ,\;\;\;\frac{\beta }{\alpha }=s, \end{array}$$

we can transform (106) as (92). It is a direct computation, so the details are omitted. Since *x*_*o*_ is arbitrarily chosen, (92) holds in *U*.

The left proofs are similar. So the details are omitted.

We have found the projective factor for each class in Theorem 11. This also gives a proof to the inverse of Theorem 11.

## 6. Singular Classes in Theorems 9 and 11

In this section, we will firstly discuss the local structures of the singular classes in Theorem 9(iii) and Theorem 9(iv), and then construct some examples for Theorem 11(iii) and Theorem 11(iv).

 Since every two-dimensional Riemann metric is locally conformally flat, we may put

(109) $$\begin{array}{c} \alpha =e^{\sigma (x)}\sqrt{{\left(y^{1}\right)}^{2}+{\left(y^{2}\right)}^{2}}, \end{array}$$

where *x* = (*x* ^1^, *x* ^2^). Since (59) is equivalent to *b* ^2^ = ||*β*||_*α*_^2^ = constant in two-dimensional case (see a simple proof in [7]), (59) holds if and only if *β* is in the form

(110) $$\begin{array}{c} \beta =\frac{be^{\sigma (x)}\left[\xi \left(x\right)y^{1}+\eta \left(x\right)y^{2}\right]}{\sqrt{\xi {\left(x\right)}^{2}+\eta {\left(x\right)}^{2}}}, \end{array}$$

where *b* = ||*β*||_*α*_ = constant. Thus (109) and (110) give all the local solutions of (59). If we put *α* and *β* in the forms (109) and (110), then we have (59); that is,

(111) $$\begin{array}{c} r_{ij}=2\tilde{\tau }\left(b^{2}a_{ij}-b_{i}b_{j}\right)-\frac{1}{b^{2}}\left(b_{i}s_{j}+b_{j}s_{i}\right), \end{array}$$

where $\tilde{\tau }$ is given by

(112) $$\begin{array}{c} 2\tilde{\tau }=\frac{\left(\xi^{2}+\eta^{2}\right)\left(\xi \sigma_{1}+\eta \sigma_{2}\right)-\xi \eta \eta_{1}+\xi^{2}\eta_{2}+\eta^{2}\xi_{1}-\xi \eta \xi_{2}}{be^{\sigma }{\left(\xi^{2}+\eta^{2}\right)}^{3/2}}, \end{array}$$

where *σ*_1_ : = ∂*σ*/∂*x* ^1^, *σ*_2_ : = ∂*σ*/∂*x* ^2^, and so forth. Further, *β* is not closed if and only if

(113) (ξ2+η2)(ξσ2−ησ1)−ξ2η1 −ξηη2+ξηξ1+η2ξ2≠0.

In particular, if we put *ξ* = *x* ^2^, *η* = −*x* ^1^, and *σ* = *c*[(*x* ^1^)^2^ + (*x* ^2^)^2^], where *c* ≠ 0 is a constant, then we have $\tilde{\tau }=0$ by (112) and (113) holds (*β* is not closed).

**Proposition 13**

Define a two-dimensional (*α*, *β*)-metric *F* on *R* ^2^ by

(114) $$\begin{array}{c} F=\frac{b\alpha^{2}+k\beta^{2}}{\sqrt{b^{2}\alpha^{2}-\beta^{2}}}, \end{array}$$

where *k*, *b* are constants with *k* ≠ −1/*b*. Then *F* is a Douglas metric if and only if *α* and *β* can be locally defined by (109) and (110), where *ξ*, *η*, and *σ* are some scalar functions on *R* ^2^. There are many choices for *ξ*, *η*, and *σ* such that *β* is not closed.

**Proposition 14**

Let *F* = *αϕ*(*s*), *s* = *β*/*α*, be a two-dimensional (*α*, *β*)-metric, where *ϕ*(0) = 1. Let *ϕ*(*s*) be given by (36) or (38) (not given by (59)). Then *F* is a Douglas metric if and only if *α* and *β* can be locally defined by (109) and (110), where *ξ*, *η*, and *σ* are some scalar functions satisfying $\tilde{\tau }=0$ in (112). There are many choices for *ξ*, *η*, and *σ* such that *β* is not closed.

Next we construct some singular examples for Theorem 11(iii) and Theorem 11(iv) which are projectively flat. One can directly verify the following two examples.

**Example 15**

Let *F* = *αϕ*(*s*), *s* = *β*/*α*, be two-dimensional (*α*, *β*)-metric, where *ϕ*(0) = 1. Let *ϕ*(*s*) be given by (36) with *k* = 0, and define *α* and *β* by (109) and (110), where

(115) $$\begin{array}{c} \xi =x^{2},\;\;\eta =-x^{1},\;\;\sigma =\left(m-\frac{1}{2}\right)\ln\left[{\left(x^{1}\right)}^{2}+{\left(x^{2}\right)}^{2}\right]. \end{array}$$

Then *F* is projectively flat with *β* being not closed.

**Example 16**

Let *F* = *αϕ*(*s*), *s* = *β*/*α*, be two-dimensional (*α*, *β*)-metric, where *ϕ*(0) = 1. Let *ϕ*(*s*) be given by (38) with *k* = 0, and define *α* and *β* by (109) and (110), where

(116) $$\begin{array}{c} \xi =x^{2},\;\;\eta =-x^{1},\;\;\sigma =\left(m-1\right)\ln\left[{\left(x^{1}\right)}^{2}+{\left(x^{2}\right)}^{2}\right]. \end{array}$$

Then *F* is projectively flat with *β* being not closed.

It might be also an interesting problem to show the local structures of the two classes of Theorem 11(iii) and Theorem 11(iv). This problem is still open.

## 7. Proof of Theorem 2

In the following proof, our idea is to choose a suitable deformation on *α* and *β* such that we can obtain a conformal form on a Riemannian space. Then using the result in [14], we can complete our proof.

Define a new Riemann metric $\tilde{\alpha }$ and a 1-form $\tilde{\beta }$ by

(117) $$\begin{array}{c} \tilde{\alpha }:=\sqrt{\xi \alpha^{2}+\eta \beta^{2}},\;\tilde{\beta }:=\beta , \end{array}$$

where

(118) $$\begin{array}{c} \xi :=\frac{{\left(1\mp b^{2}\right)}^{3}}{{\left(1\pm 2b^{2}\right)}^{3/2}},\\ \eta :=\frac{9}{8b^{2}}\left\{{\left(1\pm 2b^{2}\right)}^{3/2}-\frac{1\mp 2b^{2}+4b^{4}}{{\left(1\pm 2b^{2}\right)}^{3/2}}\right\}. \end{array}$$

Since *F* = *α* ± *β* ^2^/*α* is a Douglas metric, we have (40). By (117) and (40), a direct computation gives

(119) $$\begin{array}{c} {\tilde{r}}_{ij}=\frac{2\tau {\left(1\mp b^{2}\right)}^{2}}{{\left(1\pm 2b^{2}\right)}^{5/2}}{\tilde{a}}_{ij}. \end{array}$$

So $\tilde{\beta }=\beta$ is a conformal 1-form with respect to $\tilde{\alpha }$.

Since $\tilde{\alpha }$ is a two-dimensional Riemann metric, we can express $\tilde{\alpha }$ locally as

(120) $$\begin{array}{c} \tilde{\alpha }:=e^{\sigma }\sqrt{{\left(y^{1}\right)}^{2}+{\left(y^{2}\right)}^{2}}, \end{array}$$

where *σ* = *σ*(*x*) is a scalar function. We can obtain the local expression of $\tilde{\beta }=\beta$ by (119) and (120) (see [14]). By the result in [14], we have

(121) $$\begin{array}{c} \tilde{\beta }={\tilde{b}}_{1}y^{1}+{\tilde{b}}_{2}y^{2}=e^{2\sigma }\left(uy^{1}+vy^{2}\right), \end{array}$$

where *u* = *u*(*x*), *v* = *v*(*x*) are a pair of scalar functions such that

(122) $$\begin{array}{c} f\left(z\right)=u+iv,\;\;\;z=x^{1}+ix^{2}, \end{array}$$

is a complex analytic function.

We can express *σ* using *u*, *v*, *b* ^2^ by computing the quantity ${||\beta ||}_{\tilde{\alpha }}^{2}$. Firstly, by (120) and (121) we get

(123) $$\begin{array}{c} {||\beta ||}_{\tilde{\alpha }}^{2}=e^{2\sigma }\left(u^{2}+v^{2}\right). \end{array}$$

On the other hand, by the definition of $\tilde{\alpha }$ in (117), the inverse ${\tilde{a}}^{ij}$ of ${\tilde{a}}_{ij}$ is given by

(124) $$\begin{array}{c} {\tilde{a}}^{ij}=\frac{1}{\xi }\left(a^{ij}-\frac{\eta b^{i}b^{j}}{\xi +\eta b^{2}}\right). \end{array}$$

Now plug *ξ* and *η* into the above, and we obtain

(125) $$\begin{array}{c} {||\beta ||}_{\tilde{\alpha }}^{2}={\tilde{a}}^{ij}b_{i}b_{j}=\frac{b^{2}}{{\left(1\pm 2b^{2}\right)}^{3/2}}. \end{array}$$

Thus by (123) and (125) we get

(126) $$\begin{array}{c} e^{2\sigma }=\frac{1}{u^{2}+v^{2}}\frac{b^{2}}{{\left(1\pm 2b^{2}\right)}^{3/2}}. \end{array}$$

Finally, by plugging (126), (120), and (121) into (117), we easily get *α* and *β* given by (6) and (7), respectively, where we define *B* : = *b* ^2^. 

 The Riemann metric *α* expressed in (6) is generally not in the conformally flat form. We show another example which is expressed in a different form. Put

(127) $$\begin{array}{c} \alpha =e^{\sigma }\sqrt{{\left(y^{1}\right)}^{2}+{\left(y^{2}\right)}^{2}},\;\;\;\beta =e^{\sigma }\left(\xi y^{1}+\eta y^{2}\right),\\ \sigma =-\frac{3}{2}\ln\left[1\pm 2{\left(x^{1}\right)}^{2}\pm 2{\left(x^{2}\right)}^{2}\right]+c,\\ \xi =x^{2},\;\;\eta =-x^{1}, \end{array}$$

where *c* is a constant. Then *β* is not closed and satisfies (40) with *τ* = 0. Therefore, *F* = *α* ± *β* ^2^/*α* is a Douglas metric.

## 8. Proof of Theorem 3

This proof is similar to that in Theorem 2. In the proof of Theorem 2, we make a deformation on *α* and keep *β* unchanged. For the proof of Theorem 3 in the following, we will give a deformation on *β* but keep *α* unchanged.

Define a Riemannian metric $\tilde{\alpha }$ and 1-form $\tilde{\beta }$ by

(128) $$\begin{array}{c} \tilde{\alpha }:=\alpha ,\;\;\tilde{\beta }:=\frac{\beta }{c}, \end{array}$$

where *c* = *c*(*b* ^2^) is defined in (9), where we define *B* : = *b* ^2^. By (32) and a direct computation, we can obtain

(129) $$\begin{array}{c} {\tilde{b}}_{i\;|\;j}=\frac{2\tau \left(1+k_{1}b^{2}\right)}{c}{\tilde{a}}_{ij}=\frac{2\tau \left(1+k_{1}b^{2}\right)}{c}a_{ij}. \end{array}$$

So $\tilde{\beta }$ is a closed 1-form conformal with respect to *α*.

Now we express *α* locally as

(130) $$\begin{array}{c} \alpha :=e^{\sigma }\sqrt{{\left(y^{1}\right)}^{2}+{\left(y^{2}\right)}^{2}}, \end{array}$$

where *σ* = *σ*(*x*) is a scalar function. Then by the result in [14], we have

(131) $$\begin{array}{c} \tilde{\beta }={\tilde{b}}_{1}y^{1}+{\tilde{b}}_{2}y^{2}=e^{2\sigma }\left(uy^{1}+vy^{2}\right), \end{array}$$

where *u* = *u*(*x*), *v* = *v*(*x*) are a pair of scalar functions such that

(132) $$\begin{array}{c} f\left(z\right)=u+iv,\;\;z=x^{1}+ix^{2}, \end{array}$$

is a complex analytic function.

By the property of *u*, *v* and the fact that $\tilde{\beta }$ given in (131) is closed, it is easily seen that *u*, *v*, and *σ* satisfy the PDEs (10).

Now we determine *σ* in terms of the triple (*B*, *u*, *v*), where *B* : = *b* ^2^. Firstly by (128) and then by (130) and (131) we get

(133) $$\begin{array}{c} {||\tilde{\beta }||}_{\alpha }^{2}=b^{2}c^{-2},\;\;{||\tilde{\beta }||}_{\alpha }^{2}=e^{2\sigma }\left(u^{2}+v^{2}\right). \end{array}$$

Therefore, by (133), we get (11).

Finally, we can easily get *α* and *β* given by (9) from (11), (128), (130), and (131). 
